# Assessing Ecological Connectivity for *Loxodonta africana* Across Transfrontier Conservation Areas in Southern Mozambique

**DOI:** 10.1002/ece3.73599

**Published:** 2026-05-18

**Authors:** Francesca Romana Trezza, Sabrina Muscolino, Cornelio Ntumi, Paolo Ramoni Perazzi, Alessio Farcomeni, Zaira Valgi Macheve, João Almeida, Bob Mandinyenya, Miguel Gonçalves, Maria Pinto D'Ambanguine, Fabio Attorre, Almeida Guissamulo, Ana Gledis da Conceição, Hugo Mabilana, Gianluca Pio Zaffarano, Luca Malatesta

**Affiliations:** ^1^ Department of Environmental Biology Sapienza University of Rome Rome Italy; ^2^ Department of Biological Sciences Eduardo Mondlane University Maputo Mozambique; ^3^ Faculty of Engineering, Modeling and Simulation Centre (CeSiMo) University of the Andes Merida Venezuela; ^4^ Department of Enterprise Engineering University of Rome Tor Vergata Rome Italy; ^5^ Mozambique Wildlife Alliance Maputo Mozambique; ^6^ Scientific Services, Gonarezhou Conservation Trust, Gonarezhou National Park Chiredzi Zimbabwe; ^7^ Maputo National Park Matutuine Mozambique; ^8^ Museu de História Natural de Maputo Maputo Mozambique; ^9^ Administração Nacional das Áreas de Conservação, Rua da Resistência Maputo Mozambique

**Keywords:** African bush elephant, Circuitscape, conservation planning, ecological corridor, habitat modeling, resource selection function

## Abstract

Biodiversity loss driven by habitat degradation and fragmentation poses a critical threat to global ecosystems and demands conservation strategies that go beyond isolated protected areas. We assessed the functional connectivity of African bush elephants ( *Loxodonta africana* ) in Mozambique by combining Resource Selection Function modeling with connectivity analysis, focusing on the Great Limpopo and Lubombo Transfrontier Conservation Areas. Our results revealed that African bush elephant habitat in the region extends well beyond the boundaries of National Parks, underscoring the critical role of unprotected and other types of protected areas in maintaining functional connectivity. The Human Modification Index was the most influential variable affecting habitat suitability (72% variable importance), and many key connectivity areas intersect with peri‐urban zones and local community lands, indicating a strong spatial overlap between conservation priorities and human land use. These findings emphasize the need to develop an ecological network in the region that integrates anthropogenic pressures into conservation planning to ensure the effectiveness of ecological corridors. Synthesis and applications: Our findings identify key connectivity areas in southern Mozambique that should be prioritized for corridor management to maintain landscape‐scale connectivity. Results demonstrate that integrative conservation strategies addressing shared human–wildlife spaces and promoting cross‐border cooperation are essential. The identified corridors offer strategic entry points for inclusive governance, participatory land use planning, and sustainable management to support long‐term elephant movement and genetic exchange across this socio‐ecologically complex transboundary region.

## Graphical abstract

We present the first spatially explicit assessment of ecological connectivity for African bush elephant ( *Loxodonta africana* ) in southern Mozambique, spanning the Great Limpopo and Lubombo Transfrontier Conservation Areas. By integrating Resource Selection Function with landscape connectivity analysis, we provide spatially explicit prioritization of potential ecological corridors in a data‐limited and socio‐ecologically complex setting. This pioneering study offers both novel ecological insights and an accessible methodology to support transboundary conservation and long‐term species persistence.

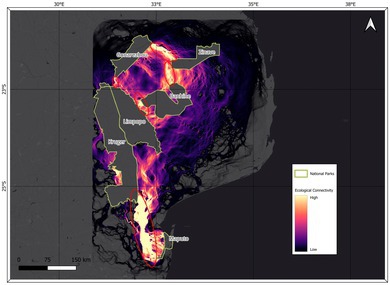

## 1 Introduction

Biodiversity loss driven by ecosystem degradation and fragmentation represents a major global challenge (Haddad et al. 2015; Venter et al. 2016), largely resulting from land conversion, infrastructure expansion, and intensifying human pressures (Ellis et al. 2010; Albers et al. 2017). These processes reduce habitat quality, isolate populations, and disrupt key ecological functions such as dispersal, recolonization, and gene flow (Fahrig 2003; Tucker et al. 2018). The Intergovernmental Science‐Policy Platform on Biodiversity and Ecosystem Services (IPBES), an international body assessing the state of biodiversity and ecosystem services, estimates that around 1 million species are currently at risk of extinction, with habitat loss and fragmentation among the leading drivers (MEA 2005; IPBES 2019). Maintaining ecological connectivity, broadly defined as the ability of organisms, genes, and ecological processes to move across the landscape (Noss 1991; Saura et al. 2018; Hilty et al. 2020), is therefore increasingly recognized as a central goal of conservation policy rather than a complementary objective.

Conservation areas remain fundamental for biodiversity protection, yet they are often too small, isolated, or subject to growing anthropogenic pressures to ensure long‐term population viability on their own (Jones et al. 2018; IUCN WCPA 2019; Maxwell et al. 2020). Most countries have failed to meet international connectivity targets: only about 10% of protected lands worldwide are structurally connected by intact habitat, and most national networks still fall short of global goals for size, representation, and connectivity (Ward et al. 2020).

Ecological connectivity is now explicitly embedded in global frameworks such as the Convention on Migratory Species, the Convention on Biological Diversity, and the Kunming–Montreal Global Biodiversity Framework, which emphasize the integration of protected areas into larger, connected ecological networks by 2030 (CMS 2020). In this context, ecological corridors are increasingly formalized as spatial units to be identified, managed, and governed with the explicit purpose of maintaining or restoring connectivity across multiple land uses (Bennett and Mulongoy 2006; Popescu et al. 2022).

Ecological connectivity can be described in structural terms, as the physical arrangement of habitat patches and barriers (Boitani et al. 2007), or in functional terms, which consider how species actually move through and respond to landscape conditions (A. F. Bennett 1998; Rudnick et al. 2012). Functional connectivity is particularly relevant for wide‐ranging megafauna whose movement decisions integrate habitat suitability, risk, memory, access to water, and avoidance of people (Cushman et al. 2018; Brennan et al. 2020; Pither et al. 2023).

Large herbivores such as the African bush elephant ( *Loxodonta africana* ; referred to by the IUCN as the African savanna elephant and hereafter as *elephant*) are widely recognized as umbrella species and landscape engineers. Maintaining their capacity to move across landscapes is essential not only for their own persistence but also for sustaining habitat continuity and ecological processes that benefit multiple taxa, including seed dispersal, vegetation dynamics, and nutrient redistribution (Roberge and Angelstam 2004; Haynes 2012; Yang et al. 2023). At the same time, they are among the most conflict‐prone species in Africa, with movements often intersecting croplands, peri‐urban areas, and community rangelands (Hoare 2015; Gross et al. 2021; Henley et al. 2023).

Southern Africa represents a flagship region for transboundary conservation initiatives. The Southern African Development Community (SADC) Transfrontier Conservation Area (TFCA) Programme, formally established in 2013, provides a policy and operational framework for enhancing ecological connectivity and promoting cooperative natural resource management across national borders. Under this framework, 18 terrestrial and marine TFCAs have been designated across the region, integrating biodiversity conservation, regional development, and peacebuilding objectives (SADC 2023). These areas aim to maintain ecological processes, such as wildlife migration, hydrological flow, and gene exchange, while fostering collaboration between governments, local communities, and private stakeholders. In Mozambique, TFCAs such as the Great Limpopo (GLTFCA) and Lubombo (LTFCA) are particularly critical for restoring connectivity for wide‐ranging species like the elephant, as approximately 76% of elephants in Mozambique, Zimbabwe, South Africa, and Eswatini regularly move across international boundaries and between protected areas (Henley et al. 2023; Mandinyenya, Cunliffe, et al. 2025; Mandinyenya, Mingione, et al. 2025). The GLTFCA and LTFCA together encompass more than 100,000 km^2^ of semi‐arid savannas, woodlands, floodplains, and coastal systems extending across Mozambique, South Africa, Zimbabwe, and Eswatini. These landscapes support some of the continent's largest remaining elephant populations but are increasingly affected by land‐use change, infrastructure development, and human settlement expansion (Chase et al. 2016; Spies 2018; National Administration of Conservation Areas 2021).

Although elephant numbers have stabilized or increased in core areas such as Kruger National Park (KNP) in South Africa and Gonarezhou National Park (GNP) in Zimbabwe, densities remain substantially lower in Mozambique. Regional estimates from the Great Elephant Census indicate densities of approximately 0.88 elephants/km^2^ in South Africa and 1.20 elephants/km^2^ in Zimbabwe, compared to only 0.10 elephants/km^2^ in Mozambique (Chase et al. 2016). These patterns are consistent with studies documenting stable or increasing populations in South Africa and Zimbabwe (Dunham 2012; Ferreira et al. 2017; Louw et al. 2021; ZimParks 2021). In contrast, many National Parks (NPs) in Mozambique have been established or rehabilitated relatively recently, Maputo NP in 2019, Banhine NP in 2001, Zinave NP in 2002, and Limpopo NP in 2001, and elephant populations, after historical declines due to the ivory trade, civil war, and land‐use policies, are still recovering. The most recent continent‐wide census estimated about 9605 elephants in the country (Chase et al. 2016), representing a substantial decline compared to historical population levels. Estimates suggest that Mozambique supported 50,000–65,000 elephants in the 1970s, declining to approximately 13,000 by 1990 because of the impacts of civil war and poaching (Ntumi et al. 2009). Although populations have shown signs of stabilization in recent decades, they remain comparatively low and fragmented across the landscape (Ntumi 2012; Booth and Dunham 2016; Thouless et al. 2016). There is thus an urgent need for spatially explicit and operationally relevant information on where ecological corridors could realistically be maintained or restored, and where connectivity is already functionally compromised by land use, governance boundaries, or social conflict (Rouget et al. 2006; Roever et al. 2013). Although the literature on elephant movement ecology and protected area networks in southern Africa is extensive, few studies have explicitly mapped functional connectivity at a transboundary scale in Mozambique, nor have they spatially identified conservation priorities linked to cross‐border movements.

Here, we assess landscape‐level potential ecological connectivity for elephants across southern Mozambique, focusing on the GLTFCA and LTFCA. We combine Resource Selection Function modeling with Circuitscape connectivity analysis, using habitat use probability as a proxy for landscape permeability. Rather than modeling individual movement paths, our goal is to provide a broad‐scale, reproducible framework applicable in data‐limited contexts. Specifically, we aim to (i) quantify habitat use probability, (ii) map potential functional connectivity between major NPs, and (iii) identify priority zones where targeted management and conflict mitigation could enable these areas to function as ecological corridors, supporting long‐term elephant movement and gene flow in southern Mozambique. The outcomes of this work are intended to provide spatially explicit, decision‐support information that can guide spatial conservation planning, land‐use management, and transboundary coordination among the institutions involved in the GLTFCA and LTFCA. By identifying areas where the landscape could potentially support elephant movement, this framework can help prioritize restoration, mitigation, and coexistence measures to maintain long‐term connectivity across southern Mozambique.

## 2 Materials and Methods

### 2.1 Study Area

The Great Limpopo Transfrontier Conservation Area (GLTFCA) spans over 100,000 km^2^ across southern Mozambique, southeastern Zimbabwe, and northeastern South Africa, with the Great Limpopo Transfrontier Park (GLTP), established on 9 December 2002, as its ecological core. Centered at latitude −22.43 and longitude 31.37, the GLTP covers 37,572 km^2^, including Limpopo National Park (LNP, Mozambique), Kruger National Park (KNP, South Africa), and Gonarezhou National Park (GNP, Zimbabwe). The GLTFCA also incorporates Banhine (BNP) and Zinave National Parks (ZNP) in Mozambique, as well as Malipati Safari Area and Manjinji Pan Sanctuary in Zimbabwe (Games 2011; Spies 2018).

The landscape consists mainly of mopane woodland, sandveld, riverine forests, miombo, and grasslands on flat terrain with mild elevation gradients (Stalmans and Wishart 2005; Ministério do Turismo 2010). The climate is subtropical to arid, with hot, wet summers and mild, dry winters. Rainfall ranges from 399 mm in BNP to 700 mm in southern KNP, with temperatures from near freezing to above 45°C (Spies 2018).

The Lubombo Transfrontier Conservation Area (LTFCA), created in 2000, spans ~10,029 km^2^ (centered at latitude −26.25 and longitude 31.88), linking ecosystems from the coastal plains to the Lubombo Mountains (Smith and Leader‐Williams 2006; Smith et al. 2008). It includes Maputo National Park (MNP, Mozambique), Tembe Elephant Park and iSimangaliso Wetland Park (South Africa), and Hlane Royal National Park and Mlawula Nature Reserve (Eswatini). MNP features coastal, terrestrial, and marine ecosystems, with sand thicket, woody grassland, sand forest, and hygrophilous grassland dominating. The terrain is flat to undulating, reaching 190 m in dunes and ridges, with a subtropical climate (26°C–30°C in wet summers, 16°C–26°C in dry winters, annual rainfall 600–1050 mm). Hydrology includes the Futi and Maputo rivers, several lakes (Piti, Xinguti, Munde), and the nutrient‐rich waters of Maputo Bay (National Administration of Conservation Areas 2021).

The GLTFCA hosts 45,000–50,000 elephants, concentrated mainly in KNP (> 30,000) and GNP (> 11,000), where densities exceed 2 elephants/km^2^ (Spies 2018; ZimParks 2021), whereas Mozambican parks maintain lower but recovering populations. LNP benefits from connectivity with KNP, whereas BNP and ZNP have relied largely on reintroductions and recovery interventions (National Administration of Conservation Areas 2022). MNP supports approximately 400–500 elephants, bolstered since 2010 by translocations and by its connection to Tembe through the Futi Corridor (National Administration of Conservation Areas 2021). Elephant movements in the region extend beyond protected area boundaries, with approximately 28% of recorded locations in Mozambique occurring outside formal conservation zones (Henley et al. 2023). Connectivity between the Great Limpopo and Lubombo TFCAs involves major parks such as KNP, LNP, BNP, ZNP, and MNP, and often occurs across human‐modified landscapes where rural communities are present (Roever et al. 2013). These movements frequently traverse human‐modified landscapes, where numerous rural communities are established in proximity to or within park boundaries. Around 1380 households are located in LNP, 4200 residents in ZNP, 3500 in BNP's buffer zone, and about 41,000 in and around MNP (Ministério do Turismo 2010; National Administration of Conservation Areas 2021). Local livelihoods depend largely on agriculture, livestock, fishing, and wild resources, making interactions with elephants particularly sensitive. Consequently, human–elephant conflict represents a major challenge in these areas (Ntumi et al. 2009; Ntumi 2012).

**FIGURE 1:**
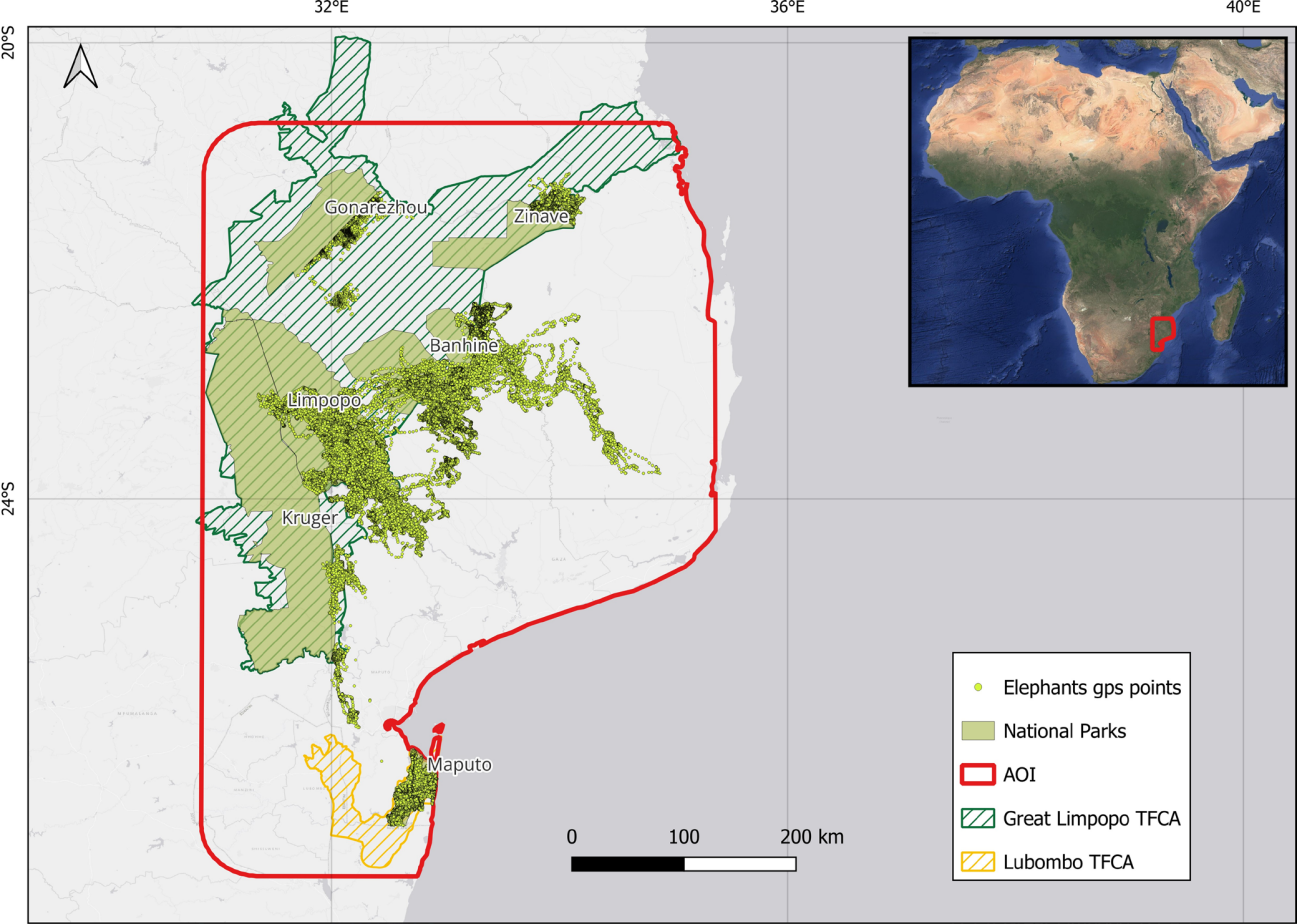
Study area showing the Great Limpopo and Lubombo TFCAs borders and main National Parks, together with the African bush elephant tracking data from Peace Parks Foundation (PPF), Mozambique Wildlife Alliance (MWA), Maputo National Park (PNAM), and Gonarezhou Conservation Trust (GCT). The area within the red polygon represents the Area of Interest (AOI), which defines the spatial extent where all analyses were conducted.

The Area of Interest (AOI) used for the analyses and for mapping ecological connectivity corresponds to the red polygon shown in Figure 1. It was generated by applying a 50 km buffer around the most peripheral presence points and restricting it to terrestrial areas, resulting in a polygon that fully encompasses all elephant tracking data. Consequently, the AOI does not represent a uniform 50 km buffer, particularly along coastal boundaries. This extent was defined to include all available GPS tracking data and all the NPs within the LTFCA and GLTFCA.

### 2.2 Model Development

We compiled an elephant telemetry dataset covering the GLTFCA and LTFCA across Mozambique and neighboring countries (see Table 1 and Figure 1). The dataset included movement data from 27 individuals monitored between 2016 and 2025, collected by multiple data providers. Additional information on each dataset is provided in Table 1; however, not all metadata were consistently available across datasets, and information such as sex of individuals, age class, transmission success, and collar performance could not be included. For more detailed metadata, readers are encouraged to contact the respective data providers directly.

**TABLE 1:** GPS tracking data were provided by Peace Parks Foundation (PPF), Mozambique Wildlife Alliance (MWA), Maputo National Park (PNAM), and Gonarezhou Conservation Trust (GCT).

Data Provider	Temporal Range	Source area	GPS Records	Number of individuals	Fix interval
Mozambique Wildlife Alliance–Maputo National Park	2022–2024	Great Limpopo and Lubombo TFCAs	98,678	7	1 h
Gonarezhou Conservation Trust	2016–2022	Gonarezhou NP	7127	12	4 h
Peace Parks Foundation	2021–2025	Great Limpopo and Lubombo TFCAs	180,935	8	4 h—30 min

*Note:* The dataset included 27 elephants monitored between 2016 and 2025 across the Great Limpopo and Lubombo Transfrontier Conservation Areas. Additional information is provided for each dataset, including temporal range, source area, number of GPS records, number of individuals, and fix interval.

Data cleaning in QGIS (2025) excluded implausible locations (e.g., positions falling within urban areas or in the ocean), resulting in a final dataset of 284,752 GPS fixes.

Five environmental variables were selected (Table 2), representing resource availability, landform, and human impact. The final variable combination was chosen on the basis of correlation analysis, ecological relevance, spatial and temporal resolution, and computational efficiency. Moreover, only open‐access datasets were considered, ensuring transparency and reproducibility.

**TABLE 2:** Environmental variables details. Overview of the five environmental variables used to model habitat use probability for elephants.

Variable	Dataset	Spatial resolution	Temporal resolution	Downloaded from
Normalized Difference Vegetation Index (NDVI)	Terra Moderate Resolution Imaging Spectroradiometer (MODIS) Vegetation Indices (MOD13Q1) Version 6.1	250 m	2016–2024 (median value)	https://developers.google.com/earth‐engine/datasets/catalog/MODIS_061_MOD13Q1#dois
Distance to water	JRC Yearly Water Classification History, v1.4	30 m	2016–2021	https://developers.google.com/earth‐engine/datasets/catalog/JRC_GSW1_4_YearlyHistory
Slope	NASA Shuttle Radar Topography Mission Digital Elevation Model	30 m	2000	https://developers.google.com/earth‐engine/datasets/catalog/USGS_SRTMGL1_003#description
Cropland	Digital Earth Africa's cropland extent map Africa 2019	10 m	2019	https://gee‐community‐catalog.org/projects/dea_croplands/#preprocessing‐for‐gee
Human Modification Index (HMI)	CSP gHM: Global Human Modification	1000 m	2016	https://developers.google.com/earth‐engine/datasets/catalog/CSP_HM_GlobalHumanModification#description

*Note:* For each variable, the table details the corresponding dataset, spatial and temporal resolution, and the source from which the data were obtained.

All environmental covariate datasets were accessed through Google Earth Engine (GEE, Tamiminia et al. 2020), clipped to the study area and exported as GeoTIFF rasters, and imported into QGIS for subsequent processing.

Normalized Difference Vegetation Index (NDVI) was used as a proxy for vegetation quality (Pettorelli et al. 2011; Didan 2021; Kaszta et al. 2021; Giliba et al. 2023). Although NDVI can be influenced by vegetation structure (e.g., higher values in woodland areas), in this study, it was treated as a statistical predictor, focusing on its relationship with elephant presence rather than its absolute ecological interpretation. Alternative vegetation indices (e.g., EVI, SAVI, NDWI) and land‐cover‐based proxies were tested during model development but did not improve model performance. NDVI was therefore retained as a parsimonious and widely used indicator of vegetation quality. Although temporally matched environmental layers could provide a more dynamic representation of local conditions, to ensure consistency with the purpose of the analysis, median values over the study period were used to represent long‐term vegetation conditions, reducing the influence of short‐term fluctuations and seasonal variability that are less relevant for identifying long‐term ecological corridors. Water availability, a key factor for elephant movement (Western 1975; De Boer et al. 2000), was represented by a distance‐to‐water layer on the basis of Pekel et al. (2016), using median values from 2016 to 2024. Slope, derived from NASA's DEM via the *ee.Algorithms.Terrain* function was included as elephants avoid steep terrain (Wall et al. 2006; Farr et al. 2007; Berti et al. 2025). Anthropogenic pressure was represented using the Human Modification Index (HMI), which integrates multiple human stressors into a single continuous metric (Kennedy et al. 2019; Wall, Douglas‐Hamilton, et al. 2021; Wall, Wittemyer, et al. 2021). Additional open‐source datasets were considered; however, these showed limited accuracy and spatial consistency across the study area. The HMI was therefore selected following multiple tests, as it provides a spatially consistent and integrated representation of anthropogenic pressure at a regional scale, and resulted in higher model performance compared to alternative datasets.

To further account for land use, a cropland layer derived from Digital Earth Africa (2022) was included as a binary variable (cropland/non‐cropland) to capture agricultural areas not explicitly represented by the HMI. Cropland is a key factor influencing elephant movement and human–wildlife interactions, as agricultural expansion often increases spatial overlap between elephants and local communities (Vogel et al. 2020).

In QGIS, each raster was reprojected, aligned to a common grid, and resampled to a spatial resolution of 1 km. Pseudoabsence points were randomly generated using the Random Points Inside Polygon tool, constraining random points generation to the AOI polygon. To avoid spatial overlap with used locations, pseudoabsence placement was restricted to areas outside a 10 km buffer surrounding each presence point, following Blake et al. (2003), who estimated the average daily displacement of ~10 km for elephants (Elith et al. 2010; Barve et al. 2011; Hazen et al. 2021). The same number of presence points (284,752) was generated for the pseudoabsences, ensuring a balanced comparison between used and available habitat.

GPS presence points were merged with pseudoabsence points to create a binary response variable (presence = 1, absence = 0). Environmental covariates were extracted at each point location in QGIS using the Sample Raster Values tool, and the resulting dataset was imported into R software (R Core Team 2024) for habitat selection modeling. Habitat selection was modeled using a Resource Selection Function (RSF) framework (Boyce and McDonald 1999; Manly et al. 2002; Johnson et al. 2006). RSFs estimate the relative probability of use of a location by comparing environmental conditions at used versus available sites. Formally, the RSF takes the form:

$$\omega \left(x\right)=\exp\left(\beta_{1}x_{1}+\beta_{2}x_{2}+\ldots +\beta_{k}x_{k}\right)$$

where $x_{1}\ldots x_{k}$ are the environmental covariates measured at each location, $\omega \left(x\right)$represents the positive quantity proportional to the relative likelihood of habitat use, whereas the exponential function ensures positivity and preserves the ranking implied by the linear predictor. We fitted a logistic RSF in R using the *rspf* function in the *ResourceSelection* package (Lele et al. 2019). A logit link was used, which models the natural logarithm of the ratio between the probability of use and the probability of non‐use as a linear function of the environmental predictors. Parameter uncertainty was assessed through nonparametric bootstrap resampling (B = 499), a procedure in which new datasets are repeatedly generated by randomly sampling the original data and the RSF is refitted on each replicate. The variability of the parameter estimates across the 499 bootstrap models provides empirical distributions from which robust standard errors and confidence intervals are derived.

Following model calibration, the RSF coefficients were applied to the environmental raster layers to generate a continuous habitat use probability map across the AOI. For each pixel, the linear predictor was computed from the fitted coefficients and the corresponding covariate values, and then converted into a probability using the logistic (expit) transformation:

$$p\left(x\right)=\frac{\exp\left(\beta_{0}+\beta_{1}\;x_{1}+\ldots +\beta_{k}x_{k}\right)}{1+\exp\left(\beta_{0}+\beta_{1}x_{1}+\ldots +\beta_{k}x_{k}\right)}$$

A population‐level modeling approach was adopted to identify general patterns of habitat use and connectivity across the study area, rather than individual‐specific strategies. This approach is widely used to derive generalizable spatial patterns and support conservation planning at regional scales (Boyce and McDonald 1999; Cushman et al. 2010). As the objective of this study is to inform large‐scale connectivity planning, a population‐level framework was considered more appropriate than individual‐based modeling.

The final habitat use probability raster was subsequently classified into nine quantile‐based classes to facilitate visualization and to highlight spatial gradients. This classification does not affect the underlying model outputs but is used solely to improve the interpretability of spatial patterns. Continuous habitat use probabilities are often classified into discrete classes for visualization (Morris et al. 2016). Model accuracy was evaluated using a confusion‐matrix–based assessment of sensitivity, specificity, and precision, from which we derived both the Area Under the Precision–Recall Curve (AUC‐PR) and the Area Under the Receiver Operating Characteristic Curve (AUC‐ROC). In the context of a use–availability RSF, the AUC‐PR quantifies how precision varies with recall and is particularly informative for assessing the model's ability to correctly identify used locations relative to available ones (Sofaer et al. 2019). The AUC‐ROC is obtained by plotting sensitivity (true‐positive rate) against 1—specificity (false‐positive rate) (DeLeo 1993), providing a threshold‐independent measure of the model's overall discriminatory capacity. Together, these metrics summarize the RSF's performance in distinguishing used from available locations across all possible probability thresholds. To compare the relative influence of predictors on habitat use, we then calculated standardized β coefficients. Since raw RSF coefficients are expressed in different units and are not directly comparable, all covariates were standardized (mean = 0, SD = 1) and the RSF was refitted. Standardized coefficients allow the effect of each variable to be evaluated on a common scale, providing a robust basis for assessing their relative importance in driving habitat selection.

### 2.3 Connectivity Analysis

To assess functional connectivity across TFCAs, we used Circuitscape 4.0 (Anantharaman et al. 2019). Circuitscape applies circuit theory to estimate functional connectivity, modeling animal movement as random walks through heterogeneous landscapes. It assumes that individuals have no prior knowledge of optimal paths and are more likely to move through areas of lower resistance (McRae et al. 2008; Shah and McRae 2008; Dickson et al. 2018).

Connectivity was estimated using the habitat use probability map generated in prior steps as the conductance surface, assuming that a higher probability of use indicates greater permeability (Chetkiewicz and Boyce 2009; Popescu et al. 2021). We ran Circuitscape in pairwise mode, defining NPs as focal nodes. All pairs of National Parks (NPs) not separated by another NP were included in the analysis. The KNP–MNP pair was excluded because KNP is directly connected to LNP; therefore, connectivity was instead assessed between MNP and LNP.

Although the LTFCA and GLTFCA include other types of protected areas and Other Effective Area‐Based Conservation Measures (OECMs), in this study we considered only NPs as focal nodes for the connectivity analysis. These areas were assumed to be fully suitable within their boundaries, as they represent the highest level of formal protection and are widely recognized as core habitats and key nodes for maintaining viable elephant populations and landscape connectivity (Roever et al. 2013; Dinerstein et al. 2020). This assumption was adopted to specifically focus the analysis on how elephants use and move through the surrounding landscape matrix, including other protected areas, OECMs, and unprotected lands. Although we acknowledge that habitat suitability within NPs is spatially heterogeneous, this was beyond the scope of the present study, which aims to identify potential corridors and connectivity constraints across the broader landscape.

Connectivity was evaluated both within the GLTFCA and between the GLTFCA and LTFCA (Table 3). Post‐processing of Circuitscape results and final maps production were performed using QGIS.

**TABLE 3:** Pairs of National Parks from the LTFCA and GLTFCA were used as focal nodes in Circuitscape, which was run in pairwise mode. In this mode, Circuitscape models current flow between each pair of focal areas independently, generating connectivity estimates that represent the most probable movement routes linking each protected area to every other one in the set.

NPs pairs analyzed	TFCAs
Gonarezhou–Zinave	Limpopo
Gonarezhou–Limpopo	
Gonarezhou–Banhine	
Zinave–Banhine	
Limpopo–Banhine	
Maputo–Limpopo	Lubombo–Limpopo

## 3 Results

### 3.1 Resource Selection Function

Correlation analysis indicated no strong multicollinearity among predictors. The highest positive correlation (0.361) was observed between the HMI and cropland, whereas the strongest negative correlation (−0.260) occurred between HMI and distance to water.

The RSF achieved good predictive performance, with AUC‐ROC = 0.8286 and AUC‐PR = 0.8003. The model coefficients are reported in Table 4. All predictors were highly significant (*p* < 0.001), with negative coefficients for slope (*β* = −0.0506, *z* = −4662.38), human modification (HMI; *β* = −20.37, *z* = −159.41), and distance to water (*β* = −0.0001138, *z* = −132.22). NDVI showed a positive association (*β* = 0.0002941, *z* = 52.20), as did cropland (*β* = 0.3605, *z* = 25.90). Standard errors were consistently small across predictors, indicating high precision in the parameter estimates.

**TABLE 4:** Parameter estimates from the logistic RSF model.

Predictor	Estimate	Std_Error	*z*_value	*p*
Intercept	−4.518	5.643	−0.801	0.423
Cropland	0.3605	0.01392	25.895	< 0.001
HMI	−20.37	0.1278	−159.414	< 0.001
NDVI	0.000294	5.64E‐06	52.202	< 0.001
Slope	−0.0506	1.09E‐05	−4662.38	< 0.001
Distance to water	−0.00011	8.61E‐07	−132.222	< 0.001

*Note:* The Estimate (*β*) represents the direction and strength of each predictor's effect on habitat use, with positive values indicating selection and negative values indicating avoidance. The Standard Error reflects the precision of each estimate, with smaller values indicating more reliable coefficients. The z‐value, obtained by dividing the estimate by its standard error, quantifies the strength of evidence for each effect. The *p*‐value indicates the statistical significance of the relationship, with values < 0.001 showing strong support for the predictor's influence on elephant space use.

Standardized coefficients are presented in Table 5. These values allow direct comparison of effect sizes and showed a clear gradient in variable influence: HMI had the largest standardized coefficient (β_std = −2.77), accounting for approximately 72% of the total standardized effect size. This was followed by distance to water (β_std = −0.50; 13%) and NDVI (β_std = 0.34; 9%). Slope (β_std = −0.16; 4%) and cropland (β_std = 0.08; 2%) exhibited smaller standardized effects, though all predictors remained statistically significant.

**TABLE 5:** Table showing standardized RSF coefficients, allowing direct comparison of the relative influence of each predictor on habitat use.

Predictor	Estimate	Std_Error	*z*_value	*p*	Relative Importance %
Cropland	0.078139	0.002782	28.09	< 0.001	2
HMI	−2.766160	0.009819	−281.70	< 0.001	72.1
NDVI	0.338296	0.00267	126.72	< 0.001	8.8
Slope	−0.158435	0.002017	−78.55	< 0.001	4.1
Distance to water	−0.496873	0.002479	−200.41	< 0.001	12.9

*Note:* Larger absolute coefficient values indicate the most important effects. The relative contribution (%) of each predictor to the total effect size was calculated on the basis of the absolute standardized coefficients, providing a quantitative measure of variable importance. HMI showed the greatest influence, followed by distance to water and NDVI, whereas slope and cropland exhibited smaller effects. All predictors were highly significant (*p* < 0.001).

**FIGURE 2:**
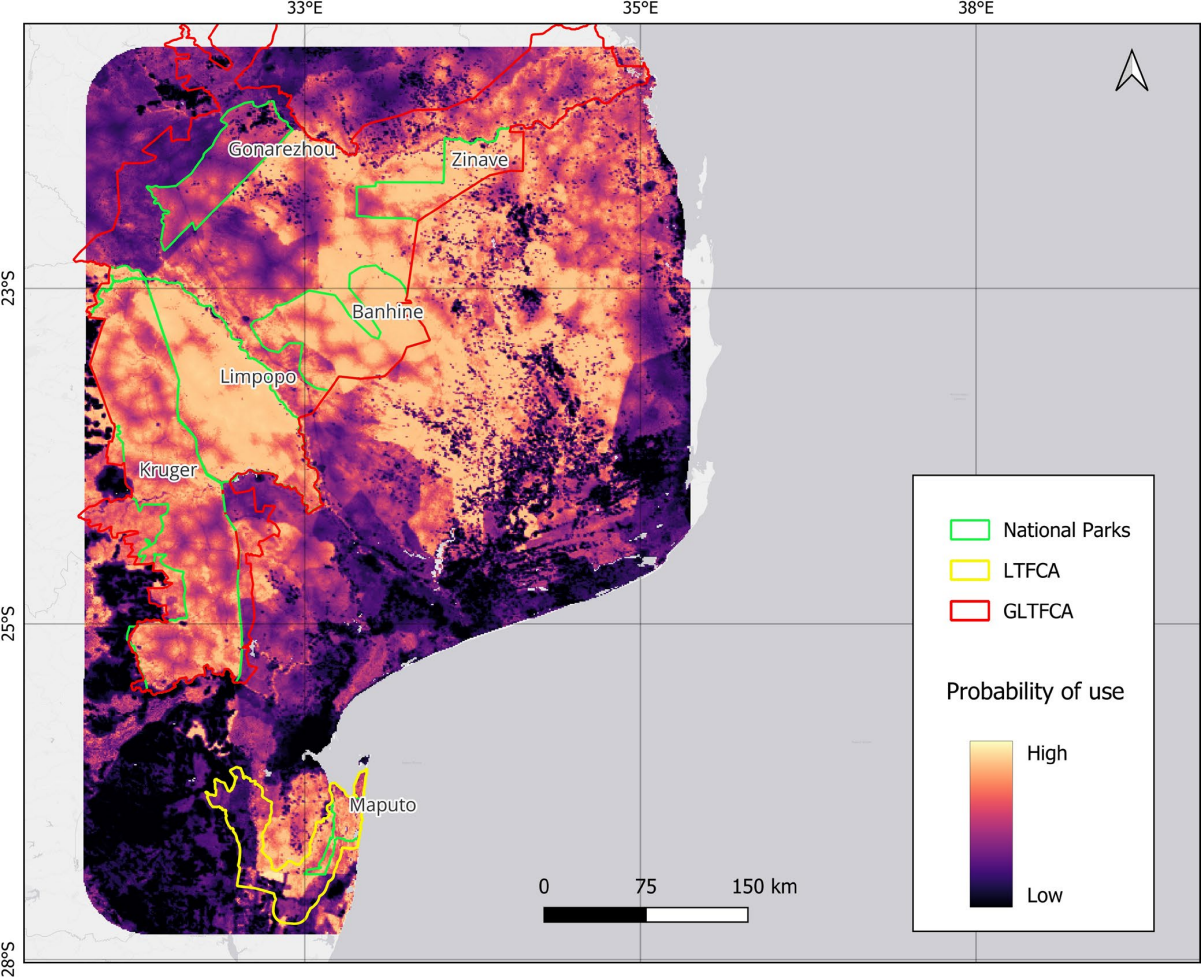
The habitat use probability map classified into nine quantile‐based classes, representing equal‐frequency intervals of predicted probability. Lower classes correspond to areas with relatively low predicted use, whereas higher classes indicate areas with a greater relative probability of elephant use. Boundaries of national parks are shown in green, whereas the extents of the Lubombo Transfrontier Conservation Area (LTFCA) and the Great Limpopo Transfrontier Conservation Area (GLTFCA) are delineated in yellow and red, respectively.

Habitat use probability values were generally higher within NPs compared to the surrounding landscape, with the exception of GNP (Figure 2). Mean values varied among parks, with the highest observed in LNP (0.0089), followed by BNP (0.0071) and ZNP (0.0067), whereas the overall mean across the study area was 0.0026. Minimum values were lowest in GNP (0.0013) and MNP (0.0042), indicating greater heterogeneity in habitat use conditions within these areas. Nevertheless, the map in Figure 2 clearly shows that many areas outside NPs have very high probability values, comparable to those within NPs.

### 3.2 Connectivity Analysis

**FIGURE 3:**
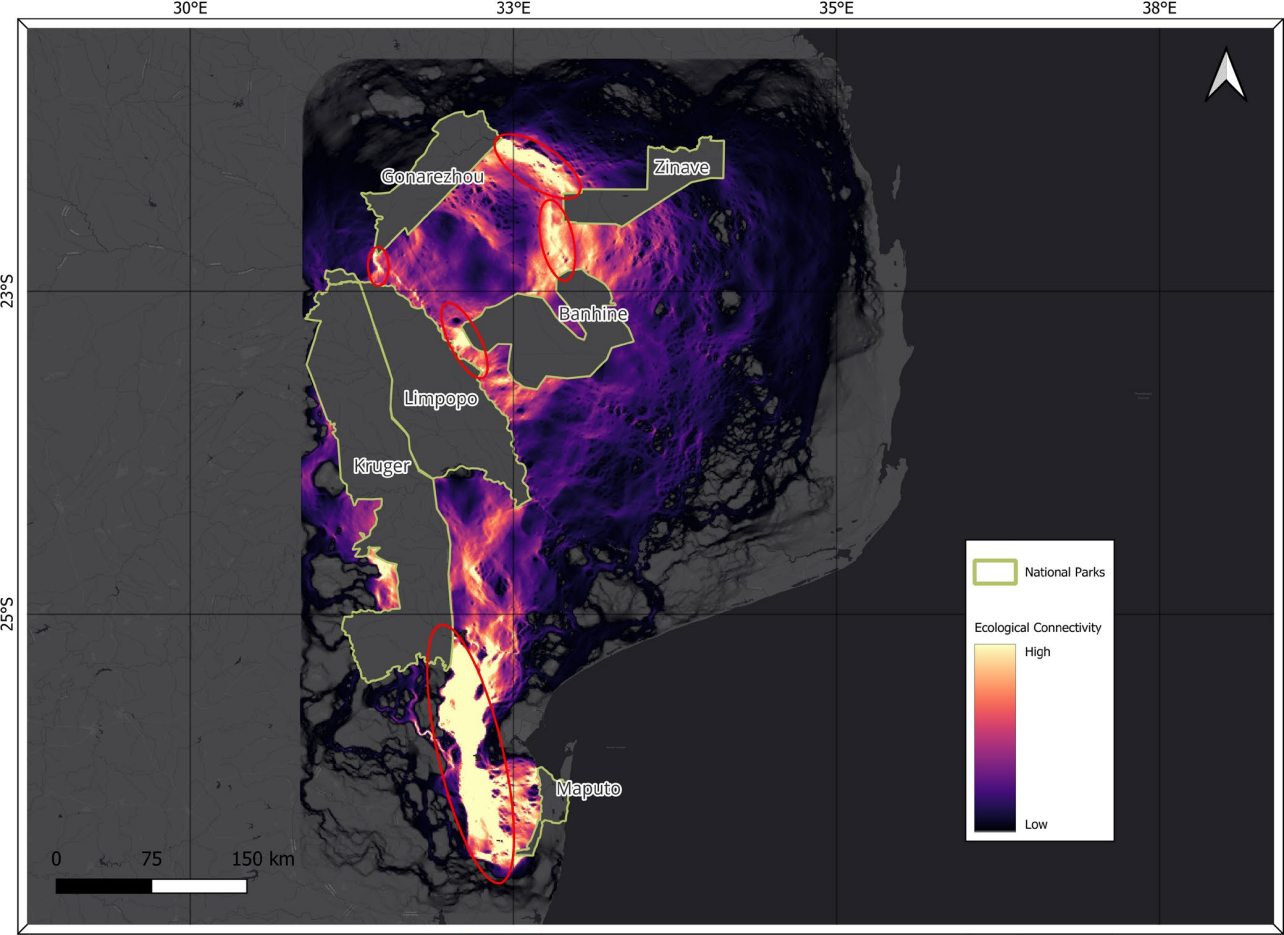
Map showing priority areas of ecological connectivity in the Lubombo and Limpopo TFCAs. The color gradient indicates relative variation, emphasizing core connectivity areas and critical bottlenecks for conservation planning (red circles). To improve the visual contrast of current flow outputs, we applied a cumulative count cut of 2%–98%. This trimming removes extreme low and high values that contribute little to the interpretation of movement patterns, allowing the main connectivity pathways to emerge more clearly in the final map.

The ecological connectivity map from the analysis is shown in Figure 3, whereas Table 6 summarizes the connectivity and resistance values for each pair of NPs.

**TABLE 6:** Mean current density and range describe the intensity and variability of potential current flow across each corridor, reflecting the relative permeability of the intervening landscape.

NPs pairs analyzed	Mean current density	Range (min–max)	Resistance
MNP–LNP	0.0011	0–0.22	2829.99
LNP–GNP	0.0005	0–0.03	86.09
GNP–ZNP	0.0005	0–0.05	77.72
LNP–BNP	0.0001	0–0.04	21.61
BNP–ZNP	0.0003	0–0.02	29.88
GNP–BNP	0.0005	0–0.04	78.94

*Note:* Resistance represents the overall difficulty for potential movement between two parks, integrating both distance and landscape heterogeneity; lower values correspond to more permeable potential corridors.

The current flow analysis revealed clear spatial patterns of ecological connectivity across the study area. A broad and continuous high‐flow corridor connected GNP and ZNP, indicating potentially strong functional linkage between the two parks. Connectivity between GNP and BNP was weaker, characterized by only moderate‐flow zones and lacking a clearly defined continuous corridor. A high connectivity corridor between BNP and ZNP was present but less pronounced, forming a large but discontinuous pathway that nonetheless suggests the potential, albeit limited, for a functional link.

Connectivity between GNP and LNP was minimal, with only a very narrow and non‐continuous corridor representing a pinch point between these two parks. The linkage between LNP and BNP revealed a well‐defined, though localized, potential corridor, characterized by consistent continuity and high connectivity values. It narrows on the LNP side and widens into a broader area near the BNP.

In the south, the pathway linking LNP and MNP showed the highest connectivity values in the study area, characterized by an extensive band of high current flow with a single notable bottleneck. KNP played a key structural role in maintaining connectivity between LNP and MNP. Connectivity weakened markedly in the area east of KNP, toward LNP, and without KNP acting as a central conduit, the two parks would exhibit considerably lower potential connectivity.

Between the LTFCA and GLTFCA, critical connectivity areas were found in Matutuine, Namaacha, Boane, Matola, Moamba, and Massingir districts, which are key to maintaining connectivity between MNP and LNP.

Within the GLTFCA, critical connectivity areas were found in northwestern Mabalane and the center of Chicualacuala District (linking LNP–BNP), and northwestern Chicualacuala (linking LNP–GNP). Between GNP‐ZNP and BNP‐ZNP, critical areas were mainly found respectively in the northwestern half of Massangena district and in the center of Chigubo District.

## 4 Discussion

This study provides key insights into the ecological connectivity of the African bush elephant within the GLTFCA and LTFCA by integrating Resource Selection Function and connectivity modeling within a reproducible framework. The resource selection model demonstrated good discriminative performance and confirms that, consistent with previous studies, RSF represents a robust approach for assessing habitat use (Boyce et al. 2002; Boyce and McDonald 1999).

Although ecological variables like vegetation quality, slope, and distance to water are widely recognized as important for elephant habitat selection (De Boer et al. 2000; Wall et al. 2006), our model revealed the overwhelming dominance of human influence (HMI), a result that is consistent with previous knowledge (Wall, Douglas‐Hamilton, et al. 2021; Wall, Wittemyer, et al. 2021; Ntukey et al. 2022; Sanare et al. 2022) and underscores the extent to which human presence now drives elephant distribution at the regional scale.

Distance to water emerged as the second most important predictor, accounting for 13% of the standardized influence on habitat use. Although this aligns with well‐established knowledge on elephants' dependence on water resources, the large gap between distance to water and HMI underscores the dominant role of anthropogenic pressure in shaping space use. Moreover, the negative correlation observed between HMI and distance to water suggests that although proximity to water is essential for elephants, it is equally important for local communities in the region, which tend to cluster along major water systems. This spatial overlap likely intensifies competition for water and contributes to reduced habitat selection in human‐dominated riparian areas and increased human‐elephant conflict (Montero‐Botey et al. 2024).

Although its influence was relatively minor compared to the dominant role of human pressure, NDVI still contributed to explaining habitat use (9%), indicating a preference for areas with higher vegetation quality, consistent with elephants' forage requirements. Similarly, slope had a limited effect (4%) but showed a negative relationship with habitat use, suggesting avoidance of steeper terrain. These patterns are consistent with previous studies, showing that elephants tend to select areas with higher vegetation quality while avoiding energetically costly or less accessible steep landscapes (Giliba et al. 2023; Berti et al. 2025). To further account for land use, a cropland layer was included to capture the potential impact of small‐scale agriculture not detected by the HMI. However, its low contribution (2%) suggests that such agriculture is not a strong determinant of habitat use and does not act as a deterrent to elephant movement, likely because fragmented and poorly fenced small‐scale agriculture allows relatively free movement across the landscape, raising concerns about potential conflict with local communities relying on these lands for subsistence farming (Vogel et al. 2020).

Overall, these results highlight the critical need to integrate detailed and accurate human pressure indicators into conservation and connectivity planning and call for the development of improved spatial proxies capable of capturing these complex human–elephant interactions more effectively.

Elephant population increases in areas like KNP and GNP (Chase et al. 2016; Edwards et al. 2024; Huang et al. 2024) represent major conservation successes, yet growing isolation from surrounding human‐modified landscapes may lead to ecological confinement and vegetation degradation. Evidence from Kruger, Gonarezhou, Hwange, and Chobe NPs shows that restricted dispersal opportunities can concentrate elephant densities, intensify browsing pressure, and reduce woodland cover (Loarie et al. 2009; Asner et al. 2016; Mandinyenya, Cunliffe, et al. 2025; Mandinyenya, Mingione, et al. 2025), emphasizing the need to maintain functional connectivity across protected area boundaries. Facilitating movement toward Mozambican lower‐density NPs could help reduce pressure on the ecosystem and maintain genetic flow (Luikart et al. 2010; Makati et al. 2025). However, from 2016 to 2025, our telemetry data showed movement only between LNP and BNP, with dispersal from high‐density to low‐density NPs limited, as well as a notable eastward displacement into unprotected areas. This latter movement, occurring outside formal conservation zones, raises questions regarding habitat use and suggests the need for further investigation to understand the ecological factors making this area attractive.

In general, the combination of connectivity values and the geographical configuration of the NPs allowed for the promising identification of potential ecological corridors across the transboundary landscape. The Circuitscape results highlight how varying levels of resistance and current density delineate areas that could support future elephant movement, as widely demonstrated in connectivity modeling studies on the basis of circuit theory. These approaches are increasingly used to identify key movement pathways and priority areas for conservation, forming a robust foundation for connectivity‐oriented planning in fragmented landscapes (McRae et al. 2008; Koen et al. 2014).

Connectivity patterns across the GLTFCA and the linkages with the LTFCA reveal a highly heterogeneous landscape, where differences in land use, human pressure, and spatial configuration of NPs shape the distribution of movement pathways. Rather than reflecting uniform connectivity, the results highlight a mosaic of areas with varying permeability, where some linkages persist despite human modification, whereas others appear constrained or fragmented (Cushman et al. 2010; Hilty et al. 2020). Overall, connectivity within the GLTFCA appears more fragmented compared to the linkage with the LTFCA, despite generally lower resistance values, suggesting that inter‐park distance and spatial configuration play a key role in shaping functional connectivity.

In this context, the corridor between MNP and LNP emerges as particularly significant, as it remains detectable despite intense human pressure and urban expansion. Additional high‐flow pathways were also observed across heavily anthropized zones near the Mozambique–Eswatini and Mozambique–South Africa borders, further highlighting the persistence of potential movement routes in regions under strong anthropogenic influence. This suggests that even heavily modified landscapes can retain a high degree of functional connectivity when natural and semi‐natural elements are sufficiently connected. The role of KNP is also critical, acting as an intermediate linkage facilitating transboundary movement between the GLTFCA and LTFCA systems. However, the fact that these linkages cross densely populated peri‐urban and agricultural areas, particularly around Maputo, Goba, Matola, and Boane, highlights their vulnerability and the need for integrated management approaches, including conflict mitigation strategies such as early‐warning systems and wildlife‐compatible agricultural practices (Hoare 2015; King et al. 2017; Gross et al. 2022; Henley et al. 2023).

Across the GLTFCA, connectivity is shaped by localized constraints, resulting in a heterogeneous network of corridors with varying levels of functionality. Rather than a uniform system, the results suggest that some linkages maintain relatively good connectivity, whereas others remain constrained or fragmented, reflecting the combined influence of land use, human pressure, and spatial configuration.

Connectivity appears comparatively stronger along the ZNP–GNP and LNP–BNP linkages, although both exhibit localized limitations such as narrowing or discontinuities that may reduce corridor functionality. These areas, therefore, represent priority zones where targeted interventions, such as habitat restoration, barrier mitigation, or the protection of key stepping‐stone habitats, could enhance connectivity and maintain movement across the landscape.

In contrast, connectivity between GNP and LNP is more constrained, with a narrow and potentially fragile linkage overlapping the Sengwe–Tshipise Corridor. This highlights the importance of this transboundary area as a strategic linkage, where improving habitat conditions and reducing anthropogenic barriers could significantly strengthen connectivity between the northern GLTFCA components.

Conversely, the GNP–BNP linkage shows weak and fragmented connectivity, consistent with findings by Mandinyenya, Cunliffe, et al. (2025); Mandinyenya, Mingione, et al. (2025), suggesting that this area may not currently support regular elephant movement or gene flow (Nzombane et al. 2025). In such cases, further investigation at finer spatial scales is needed to identify the local factors limiting connectivity and to assess whether restoration actions are feasible or whether alternative linkage strategies should be considered.

From a management perspective, these results emphasize the importance of moving beyond the protection of core areas to actively managing the surrounding matrix. Restoration efforts, barrier mitigation, and the protection of stepping‐stone habitats will be essential to maintain or re‐establish functional connectivity, particularly in fragmented landscapes. More broadly, identifying and strengthening key linkage zones while addressing socio‐ecological constraints will be critical to ensure long‐term connectivity across the GLTFCA and its linkages with the LTFCA (Cushman et al. 2010; Sawyer et al. 2011).

Several examples demonstrated practical approaches to ecological corridor implementation. Community‐managed conservancies, conservation leases, and NGO‐government partnerships have shown varying success (G. Bennett 2004; Parsons et al. 2025). The Simalaha Conservancy (Chobe NP in Botswana and Kafue NP in Zambia) restored movement and reduced poaching through community ownership. In Kenya, Kimana protected 8000 ha via leases but faces sustainability challenges. In the Albertine Rift, NGO‐supported corridors lacked legal standing and broader connectivity. Tanzania's Ruipa Corridor is hampered by weak governance and funding (Hilty et al. 2020). These examples show that success depends on legal clarity, inclusive governance, cross‐sector coordination, and long‐term funding. In this context, our study provides a spatially explicit, continuous connectivity surface that identifies priority areas for maintaining or restoring ecological linkages across the region. By highlighting where corridors are most viable and where they are most vulnerable, this work aims to support decision‐making and spatial conservation planning processes, offering an evidence‐based foundation for designing, implementing, and safeguarding ecological corridors in the GLTFCA and LTFCA landscape.

Limitations of this study include the reliance on telemetry data from 27 individuals, which may not capture the full variability of movements across age groups, sexes, social structures, or seasonal dynamics. Moreover, the adoption of a population‐level modeling approach may mask individual variability in resource selection and movement behavior, as elephants are known to exhibit heterogeneous and context‐dependent strategies. Although this approach allows for the identification of general spatial patterns relevant for regional‐scale conservation planning, it may overlook individual‐level differences in habitat use. Future work could therefore explore individual‐level responses and assess model performance across individuals to better capture this variability.

Additionally, the use of habitat use probability as the Circuitscape conductance layer, although methodologically efficient, assumes a direct correlation between habitat use probability and permeability, an oversimplification that overlooks ecological and behavioral drivers such as matriarchal leadership, culturally transmitted routes, physical barriers, and seasonal resource variability.

Further limitations are related to the environmental datasets used. Some layers, such as the water distribution map, do not include smaller or ephemeral waterholes that may be highly relevant to elephants. Similarly, the cropland dataset used in this study is derived from global products. Although a visual assessment of its accuracy was conducted by overlaying the dataset with high‐resolution satellite imagery for the study area and period, confirming a generally good representation of major agricultural areas, it may not fully capture small‐scale and heterogeneous agricultural systems typical of the region, where land use is often fragmented and dynamic. In addition, cropland is notoriously difficult to map accurately at a global scale, which may affect its representation in the model.

Moreover, it was not always possible to maintain full temporal consistency between spatial layers and GPS tracking data, as equivalent datasets were not available for the same year across all variables. This temporal mismatch may introduce uncertainty, as elephant movement decisions are influenced by short‐term environmental variability that is not fully captured by temporally aggregated datasets. The use of remotely sensed predictors at medium spatial resolution also introduces uncertainty in representing fine‐scale habitat features and movement constraints.

Our modeling approach therefore aims to identify potential key connectivity areas rather than representing realized routes. For this reason, the study should be regarded as a first regional‐scale assessment providing a broad spatial reference of habitat connectivity across the study area. Future efforts should aim to integrate higher‐resolution and temporally matched environmental layers, together with expanded telemetry datasets, to better capture the dynamics of ecological processes underlying elephant movement. We also recommend establishing a cross‐institutional, transboundary data‐sharing framework to enhance both the predictive power and conservation relevance of connectivity modeling in this landscape.

Functional ecological corridors rely on strong political commitment, effective cross‐border cooperation, integration into land‐use planning, active engagement of local communities, sustainable long‐term funding, and consistent ecological monitoring. Although this study focused on the African bush elephant, applying these principles through science‐based conservation planning can support broader ecological processes and benefit multiple species. This approach would enhance the GLTFCA and LTFCA as a truly interconnected ecological network, safeguarding long‐term ecosystem functions and fostering human–wildlife coexistence in one of Africa's most biodiverse regions.

## Author Contributions

**Francesca Romana Trezza:** conceptualization (lead), data curation (equal), formal analysis (equal), methodology (lead), writing – original draft (lead), writing – review and editing (lead). **Sabrina Muscolino:** data curation (equal), formal analysis (equal), methodology (equal), writing – original draft (equal). **Cornelio Ntumi:** conceptualization (equal), methodology (equal), writing – original draft (supporting). **Paolo Ramoni Perazzi:** data curation (equal), formal analysis (equal), methodology (equal), writing – original draft (equal). **Alessio Farcomeni:** formal analysis (lead), methodology (equal). **Zaira Valgi Macheve:** investigation (supporting), methodology (supporting). **João Almeida:** data curation (equal). **Bob Mandinyenya:** data curation (equal). **Miguel Gonçalves:** conceptualization (supporting), data curation (equal). **Maria Pinto D'Ambanguine:** data curation (equal). **Fabio Attorre:** conceptualization (equal), funding acquisition (equal), methodology (equal), writing – original draft (supporting). **Almeida Guissamulo:** conceptualization (supporting). **Ana Gledis da Conceição:** conceptualization (supporting). **Hugo Mabilana:** conceptualization (supporting). **Gianluca Pio Zaffarano:** data curation (supporting), project administration (supporting). **Luca Malatesta:** conceptualization (equal), data curation (supporting), formal analysis (supporting), methodology (supporting).

## Funding

This work was supported by Agenzia Italiana per la Cooperazione allo Sviluppo, COREBIOM–AID: 12042.

## Conflicts of Interest

The authors declare no conflicts of interest.

## Data Availability

The Google Earth Engine code used to download environmental data is available at the following link: https://code.earthengine.google.com/6b37541e670d0d768d362493db5f9f0e. The R script, resulting habitat use probability, and connectivity datasets are available in this Google Drive: https://drive.google.com/drive/folders/133P2l2ILNI2tNYt1Sxv3l4PMJ_aZ7WNA?usp=sharing. The elephant telemetry data could not be shared together with the code, as they are owned by the respective institutions and concern a sensitive species. Access to these data can only be granted upon formal request addressed to each institution.
